# Mutations in coral soma and sperm imply lifelong stem cell renewal and cell lineage selection

**DOI:** 10.1098/rspb.2022.1766

**Published:** 2023-01-25

**Authors:** Elora H. López-Nandam, Rebecca Albright, Erik A. Hanson, Elizabeth A. Sheets, Stephen R. Palumbi

**Affiliations:** ^1^ Biology Department, Hopkins Marine Station of Stanford University, Pacific Grove, CA 93950 USA; ^2^ Institute for Biodiversity and Sustainability Science, California Academy of Sciences, San Francisco, CA 94118, USA

**Keywords:** germline evolution, mutations, adaptation, stem cells, reproduction, genomics

## Abstract

In many animals, the germline differentiates early in embryogenesis, so only mutations that accumulate in germ cells are inherited by offspring. Exceptions to this developmental process may indicate other mechanisms have evolved to limit the effects of deleterious mutation accumulation. Stony corals are animals that can live for hundreds of years and have been thought to produce gametes from somatic tissue. To clarify conflicting evidence about germline-soma distinction in corals, we sequenced high coverage, full genomes with technical replicates for parent coral branches and their sperm pools. We identified post-embryonic single nucleotide variants (SNVs) unique to each parent branch, then checked if each SNV was shared by the respective sperm pool. Twenty-six per cent of post-embryonic SNVs were shared by the sperm and 74% were not. We also identified germline SNVs, those that were present in the sperm but not in the parent. These data suggest that self-renewing stem cells differentiate into germ and soma throughout the adult life of the colony, with SNV rates and patterns differing markedly in stem, soma and germ lineages. In addition to informing the evolution of germlines in metazoans, these insights inform how corals may generate adaptive diversity necessary in the face of global climate change.

## 1. Introduction

In 1889, Weismann proposed that germ and somatic tissues serve starkly different functions: germ cells protect heritable information and pass it on to the next generation, while somatic cells perform the functions necessary to keep an organism alive but do not contribute to the genetic makeup of the organism's offspring [1]. This would explain why mutations that accumulate in somatic tissues during an animal's lifetime—including those that cause cancer—are not inherited by that animal's offspring. Instead, only mutations in germ cells, which undergo fewer cell divisions and have lower mutation rates, are inherited [2,3]. Since Weismann, embryonic germ-soma separation has been shown in vertebrates and many other animal taxa, but not in plants or in some animal groups, including cnidarians, sponges, tunicates and platyheminths [4,5].

Potential animal exceptions to Weismann's Germ Plasm Theory are intriguing because they may have novel mechanisms to reduce the number of deleterious mutations inherited by sexually produced offspring [6]. Moreover, such exceptions may signal the potential existence of stem cell lineage types not seen in vertebrates. For example, the model cnidarians *Hydra* and *Hydractinia* possess interstitial stem cells, denoted i-cells, that can differentiate into both germ and soma during adult life [7,8]. A few models have hypothesized how heritable post-embryonic mutations may affect the gamete pool [9–11], but there are very few datasets on the pattern of somatic mutations and their inheritance in long-lived animals [12,13].

Clonal, colonial corals can live for hundreds to thousands of years, may or may not senesce, and have long been thought to generate gametes from the somatic cells of clonal polyps [14]. Coral colonies accumulate somatic mutations at a rate similar to noncancerous human tissues [12]. If these mutations are inherited by the coral's gametes, they may increase the heritable mutational load of these animals. Some previous studies identified putative somatic mutations in the gametes or juvenile offspring of mutant parents [15,16], but others have reported absence of somatic mutations in the gametes [17]. These studies tracked few mutations, ranging from 9 to 170, and none detected germline mutations in gametes or offspring. Only one verified that their putative mutations were not polymerase chain reaction or sequencing error [17]. By contrast, here we evaluated thousands of mutations by sequencing full genomes from multiple branches of multiple colonies. We accounted for sequencing error with technical replicates for each sample. We identified germline variants in the sperm as well as post-embryonic variants in the parent. The data reject the hypothesis that somatic cells give rise to germ cells in corals, but also reject the hypothesis that corals possess embryonic germline differentiation. Rather, we show that both parent tissue and sperm arise from a common stem cell lineage that proliferates and differentiates throughout the long lives of these animals. Our data do not indicate that somatic mutations are inherited by germ cells, but that mutations identified in both soma and germ cells are a result of inheritance from a common progenitor stem cell lineage. This is a significant departure from what the two previous hypotheses were (either that somatic mutations get inherited by the sperm, or that the development of germline ends at the embryonic stage). We also show how the different types of mutations present in each cell lineage may be owing to differing levels of selection on different cell types, within a single coral colony.

## 2. Material and methods

### (a) Sample collection

**Figure 1.**
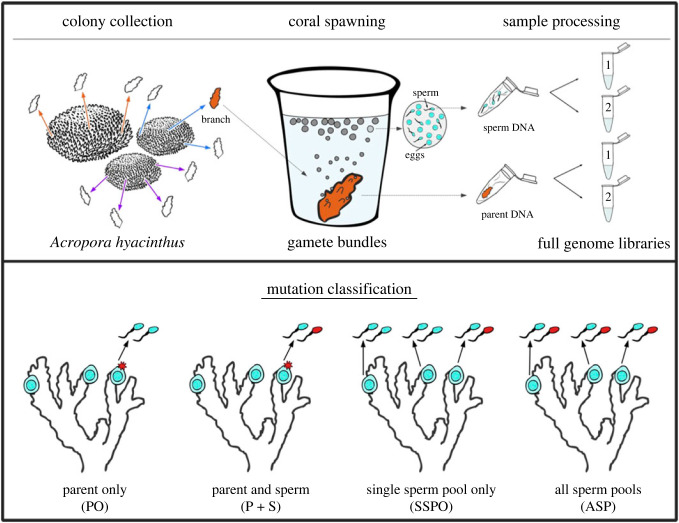
Data collection (top) and mutation classification (bottom). Top: 20 min prior to spawning, 3–4 branches were broken off of three parent colonies and placed into individual cups of seawater, for a total of 10 branches in cups. Branches then released eggs and sperm into each cup, and sperm was collected from the cup. Both the sperm pool and the parent samples were stored in RNAlater and frozen. Genomic DNA was extracted from each parent branch and sperm pool (see Methods). For each genomic DNA extraction we constructed two full genome libraries (see Methods) for technical replication. Mutation classifications (bottom, left to right): 1) a mutation unique to a single branch of the colony, but the sperm from the branch does not share the mutant genotype (parent only, PO); 2) a mutation unique to a single branch of the colony, and the sperm from the branch shares the mutant genotype (parent and sperm, P + S); 3) a mutation unique to just one sperm pool in the colony, not shared by other sperm pools or the parent branches (single sperm pool only, SSPO); and 4) a mutant genotype shared by all sperm pools from a particular colony, but none of the parent branches in that colony (all sperm pools, ASP). Figure by Shayle Matsuda.

Gravid coral colonies of *Acropora hyacinthus* were collected in Palau (Bureau of Marine Resources permit number RE-19-07 and CITES permit PW19-018) in February 2019 and transported to the Coral Spawning Laboratory at the California Academy of Sciences where they were kept on a Palauan lunar and day/night cycle until spawning, with methods adapted from [18]. Colonies were monitored for spawning activity on nights 6–9 after the simulated full moon in March 2019 (from 27 March to 30 March 2019). Prior to spawning, pliers were used to break off 2–3 cm branches that were ‘set’, or showed visual signs of impending gamete release: three branches from each of two colonies, and four branches from a third. Each branch was placed in a labelled 5 ml vial of seawater where they spawned approximately 20 min later (figure 1; electronic supplementary material, figure S1). After the gamete bundles were released, they were gently transferred to labelled 1.5 ml tubes and left to passively dissociate into eggs and sperm. Dissociation occurred over the course of 30–45 min. Upon dissociation, eggs were removed via pipet, leaving a concentrated sperm pool. Each concentrated sperm pool was pipetted into a 1.5 ml tube of RNAlater. Each coral branch was placed in a 5 ml tube of RNAlater. Sperm pools in RNAlater were stored at −20°C and coral branches in RNAlater were stored at −80°C until time for DNA extraction.

### (b) DNA extraction and library preparation

For each coral branch, the top layer of tissue was scraped from the coral skeleton with a razor blade. DNA was then extracted from tissue using the NucleoSpin Tissue Mini kit columns and corresponding protocol for extraction from animal tissue (Macherey-Nagel, Duren, Germany). For each sperm pool, the tube containing RNAlater and sperm was vortexed vigorously, then 200–400 μl of the sperm solution was pipetted out and mixed with 2× volume of deionized water. The sperm pools were then centrifuged for 3 min at 13 000 rpm. The supernatant was pipetted off, leaving just the pelleted sperm at the bottom of the tube. DNA was extracted from sperm pellets using the same Macherey-Nagel NucleoSpin Tissue Mini kit columns and protocol as the parent tissue. Nextera full genome libraries were generated using a modified, low-volume protocol optimized for coral DNA (electronic supplementary material, methods). We constructed two replicate libraries for each DNA extraction (figure 1). Libraries were sequenced first on an iSeq 100 for quality control and then on a NovaSeq 6000 S4 at the Chan-Zuckerberg Biohub Sequencing facility in San Francisco, CA, USA.

### (c) Reference genome assembly

In May 2020 we collected sperm from an additional *A. hyacinthus* colony for the construction of a high quality *A. hyacinthus* reference genome assembly. This colony originated in Palau and spawned at the California Academy of Sciences, where the sperm was collected. Sperm was collected by pipetting, then it was rinsed and spun in seawater three times at 13 000 rpm for 3 min each spin (following methods from [19]). The cleaned sperm pellet was then flash frozen in liquid nitrogen. The frozen sperm pellet was shipped to Dovetail Genomics (Scott's Valley, CA, USA) for DNA extraction, sequencing, and genome assembly. The initial *de novo* assembly was produced through a combination of Illumina short-read sequencing and PacBio long-read sequencing. Proximity ligation was achieved with Dovetail Omni-C Technology, which uses a sequence-independent endonuclease approach to chromatin fragmentation. The final genome assembly is made up of 908 scaffolds, of which 14 represent full chromosome-length scaffolds, the same number of chromosomes as is in the *Acropora millepora* genome [20]. The remaining 894 scaffolds are fragments of DNA that could not be assigned to a particular chromosome. The complete assembly is 446 422 234 nucleotides (446 Mbp), with *N*_50_ = 26 527 962 nucleotides.

### (d) Reference genome annotation

Genome annotation was performed using MAKER2 [21] in a *de novo*, iterative approach based on https://gist.github.com/darencard/bb1001ac1532dd4225b030cf0cd61ce2. Transcriptome evidence from *A. hyacinthus* [22] (and https://matzlab.weebly.com/data–code.html), *A. millepora* [20,23] and *Acropora tenuis* (https://matzlab.weebly.com/data–code.html) was provided for the initial round of annotation. Additionally, proteome evidence from *Acropora digitifera* [24] and *A. millepora* [20] was used for the first round. Genome wide repeat families were annotated by RepeatModeler2.0.1 [25] and used as evidence for the initial round. The ab initio gene predictors AUGUSTUS v. 3.2.3 [26] and SNAP [27] were trained with the gene models annotated by the previous round of annotation. The second round was then conducted with these trained prediction models along with repeat, transcript and protein evidence annotated during the previous round. A third round of annotation was then performed following the same procedures as round two. Following the final round, the completeness and quality of the annotated transcriptome was assessed with BUSCOv5 [28] and the OrthoDB v10 [29] eukaryota and metazoan datasets. The BUSCO score against the metazoan dataset was 71.3% complete, 13.6% fragmented and 15.1% missing (electronic supplementary material, table S3).

### (e) Read mapping and single nucleotide polymorphism calling

Adapters were trimmed from reads using Trimmomatic v. 0.39. Trimmed reads were mapped to the *A. hyacinthus* v1 genome using hisat2 with the parameters –very-sensitive –no-spliced-alignment. Duplicate reads were removed with Picardtools MarkDuplicates. Haplotype calling was performed with the Genome Analysis Toolkit v. 4.1.0.0 Haplotypecaller tool [30]. We combined genomic variant call format files (GVCFs) from the same coral colony into a multi-sample GVCF using CombineGVCFs. Joint genotype calling was then performed on each mutli-sample GVCF using GenotypeGVCFs with the option—all-sites to produce genotypes for both variant and nonvariants sites [31]. The genotype-called multi-sample variant call format files (VCFs) were filtered with SelectVariants to filter files by depth, with minimum depth and maximum depth determined by a Poisson distribution of the average depth for a given sample, with *p* < 0.0001 [32]. The filtered files resulting from these steps were considered the ‘callable’ regions of the genome and were used as the denominator for mutation frequency calculations. We filtered for just biallelic single nucleotide polymorphisms (SNPs) using VCFtools. For the complete read mapping and SNP calling pipeline see https://github.com/eloralopez/CoralGermline.

### (f) Identifying post-embryonic single nucleotide variants

Single nucleotide variants were identified from the genotyped colony VCFs using custom Python3 and R scripts (https://github.com/eloralopez/CoralGermline). Putative post-embryonic SNVs were identified by comparing the parent branch genotype calls from a given colony. A SNP was called a putative post-embryonic single nucleotide variant (SNV) if the SNP: (i) appeared in just one branch of the colony, and (ii) the SNP had the same genotype call in both replicate libraries from that mutant branch (figure 1*c,d*). Comparing technical replicates was highly successful at reducing noise from sequencing error, eliminating over 90% of putative SNVs that would have been identified using the same pipeline and filters without technical replicates (electronic supplementary material, figures S2–S4).

Germline mutations were identified by comparing the sperm genotype calls from a given colony. A SNP was called a single sperm pool only (SSPO) SNV if it: (i) appeared in just one sperm pool spawned by the colony, (ii) the SNP had the same genotype call in both replicate libraries from the sperm pool, and (iii) the genotype in the mutant sperm pool did not match the genotype of the parent branch that spawned it (figure 1). Alternatively, we called an SNP a putative all sperm pools (ASP) SNV if the SNP: (i) appeared in every replicate library from every sperm pool spawned by the colony, and (ii) the genotype in the sperm pools did not match the genotypes of any of the parent branches in that colony (figure 1).

### (g) Classifying putative single nucleotide variants

Once we had generated a set of putative somatic and germline mutations, we classified each SNV as either a gain of heterozygosity (GoH), where the mutant genotype is a novel heterozygous SNV, or loss of heterozygosity (LoH), where the mutant is homozygous at a site for where the rest of the samples are heterozygous and classified the directionality of the change (A to T, etc.) as described in [12].

### (h) Final filtering of putative single nucleotide variants to arrive at final set of single nucleotide variants

The final filtering step was to eliminate putative mutations that had been classified GoH if the putatively mutant allele had been seen before in any of the other libraries. This step was necessary because the filtering threshold to call a heterozygote was 10%. We set the threshold to 10% with the assumption that this would filter out SNVs that were mosaic within a single branch; the idea being that at lower variant allele frequency (VAF) the SNV may be new enough that it would not be well-mixed within a single parent branch; this was based on the lower VAFs (less than 8%) found in mosaic human organs [33]. This means that if the putatively mutant allele was present at less than 10% allele frequency in other samples, it was not truly a mutant, but present in the colony at low levels. Similarly, we eliminated putative mutations classified as LoH if the mutant sample was heterozygous at a level less than 10%. The mutations that passed this filter is the final set of mutations upon which all subsequent analyses were performed.

### (i) Are parent single nucleotide variants shared by their sperm pool or not?

Once we arrived at a set of somatic mutation with all filters applied, we checked to see if the genotype of the mutant parent matched the genotype of the sperm pool that came from that branch. GoH SNVs were considered shared if one or both genotypes of the two sperm pool replicates spawned by the mutant branch matched the mutant genotype, as this indicated that the mutant allele was present in the sperm pool, and not shared if neither of the genotypes of the two sperm pool replicates spawned by the mutant branch matched the mutant genotype (figure 1; electronic supplementary material, figure S5). LoH SNVs were considered shared if both genotypes of the two sperm pool replicates spawned by the mutant branch matched the mutant genotype, as this indicated that the heterozygous state had been lost in the sperm pool, and not shared if one or neither of the genotypes of the two sperm pool replicates spawned by the mutant branch matched the mutant genotype, as this indicated that the sperm pool retained the heterozygosity that the parent branch had lost (figure 1).

### (j) Designating single nucleotide variant effects on codons

We classified each mutation by the genomic region (intron, exon, etc.) it fell in and, if it fell in a coding region, the type (synonymous, missense) using the program snpEff [34] configured with the *A. hyacinthus* version 1 genome. We calculated the rate of missense and synonymous SNVs per bp in the coding region by dividing the number of SNVs by the total callable coding region. We calculated the average per cent of coding SNVs that were missense per sample for three categories: parent only (PO), parent and sperm (P+S) and SSPO. There were no coding SNVs in the ASP category (electronic supplementary material, figure S6).

### (k) Selection on mutations

To determine whether the percent of missense coding SNVs in the PO, P+S and SSPO categories were significantly different from what would be expected under neutrality, we estimated the per cent missense sites across the full *A. hyacinthus* proteome using a custom Python script that counts how many of each type of amino acid are present in the full proteome (https://github.com/eloralopez/CoralGermline).

### (l) Single nucleotide variant rates

To find the average frequency per nucleotide of somatic mutations unique to a given branch in a coral colony, we divided the number of SNVs in a sample by the number of total callable nucleotides sequenced for that sample (electronic supplementary material, table S1):

$$\mathrm{no}.\;\mathrm{SNVs}\;\mathrm{per}\;\mathrm{Mbp}=\left(\frac{\mathrm{no}.\;\mathrm{SNVs}}{\mathrm{callable}\;\mathrm{region}(\mathrm{bp})}\right)\times 1\;000\;000.$$

The callable genome sizes were 1.2 × 10^8^ bp, 1.2 × 10^8^ bp and 0.80 × 10^8^ bp and the callable coding region sizes were 1.3 × 10^7^ bp, 1.3 × 10^7^ bp and 0.91 × 10^7^ for each of the three colonies, CA56, CA60 and CA65, respectively (electronic supplementary material, table S1).

## 3. Results

To clarify the inheritance of mutations and the presence of germline-soma distinction in *A. hyacinthus*, we removed branches from soon-to-spawn adult coral colonies and placed them into individual cups of seawater (electronic supplementary material, figure S1). Each branch released gamete bundles into its respective cup 20 min later (figure 1; electronic supplementary material, figure S1b). We extracted DNA from each branch and each sperm pool, then constructed two replicate full genome libraries from each DNA extraction (figure 1*b*). To be verified, a SNV had to be present in both replicate libraries of a given sample. The technical replicates eliminated over 90% of putative SNVs that would have been called if we had used one library per sample, although the exact number varied by SNV category (electronic supplementary material, figures S2 and S3).

We identified four different types of SNVs: those that were unique to the polyps from a single parent branch in a colony but were not detected in the sperm from that branch (PO); those that were found in just a single parent branch in a colony and were also shared by the sperm from that branch (P + S); those that were unique to a single sperm pool in a colony and not present in any branch of the colony (SSPO); and those that were shared by all sperm pools in a colony but had never been seen in the polyps from any branch (ASP) (figure 1).

We assayed nine parent polyp samples, and the respective sperm pools for seven of those samples, across three different colonies. The average depth of coverage across the genome was 40.6 ± 3.1 (1 s.e.m) for the parent polyp libraries and 65.2 ± 6.9 (1 s.e.m) for the sperm pool libraries (electronic supplementary material, table S1). Across the full dataset we identified 2356 SNVs. All but one of these SNVs were at unique sites, indicating that the SNVs called were not a result of consistent mapping error or bias (electronic supplementary material, table S2). Each SNV was classified as a GoH, in which the aberrant sample was a new heterozygote and all others were homozygous, or an LoH, in which the aberrant sample was homozygous and the other samples were heterozygous.

**Figure 2.**
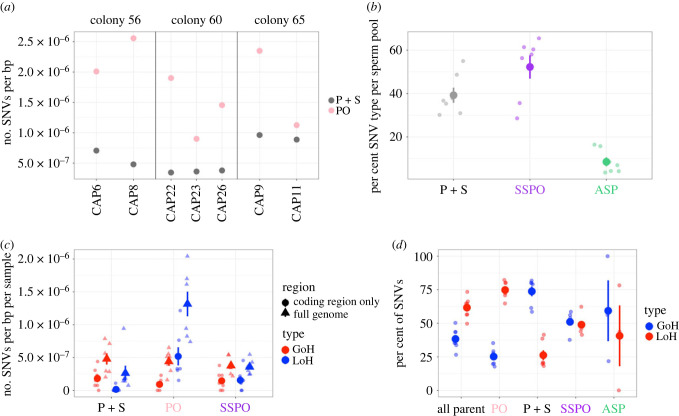
SNV rates and proportions across different classifications. (*a*) The rate of SNVs per bp for two SNV types: shared by parent and sperm (grey) and found in the parent only (yellow) for the seven parent-sperm pairs from the three colonies. (*b*) The average percentage of SNV type (parent and sperm, P + S), (parent only, PO) and all sperm pools (ASP) found in each sperm pool sample (*n* = 7). (*c*) The average rate of SNVs per bp per sample (*n* = 7) across the full genome and for the coding regions only, for three SNV types: P + S, PO, and single sperm pool only (SSPO). (*d*) The average percentage of SNVs that were GoH and LoH for each of the four SNV types found in each sample (*n* = 7). For (*b*), (*c*) and (*d*), the mean for each category is shown as a large point with error bars extending out; error bars represent ±1 s.e.m. Each individual data point (*n*= 7 for P + S, SSPO, PO, and all parent, *n* = 3 for ASP) is shown as a smaller point for each category.

We identified 146–351 post-embryonic SNVs per parent branch (electronic supplementary material, table S1). The rate of SNVs unique to a single parent branch, but not found its sperm pool (PO) ranged from 0.90–2.55 × 10^−6^ SNVs bp^−1^ branch^−1^, with an average rate of 1.76 ± 0.23 × 10^−6^ (1 s.e.m.) SNVs bp^−1^ branch^−1^ (figure 2*a*). The rate of SNVs unique to a single parent branch, and shared by its respective sperm pool (P + S) ranged from 0.34–0.96 × 10^−6^ SNVs bp^−1^ branch^−1^, with an average rate of 0.59 ± 0.1 × 10^−6^ (1 s.e.m.) SNVs bp^−1^ branch^−1^ (figure 2*a*). On average, the rate of PO SNVs was 3.4 times higher than the rate of P+S SNVs found in a given branch. On average, 25.7% ± 3.7% (1 s.e.m.) of SNVs found in a parent branch were P+S SNVs, while 74.3% ± 3.7% (1 s.e.m.) post-embryonic SNVs in a branch were PO SNVs. These findings contradict the hypothesis from the Germ Plasm Theory that post-embryonic mutations would not be found in the sperm at all. They also contradict the common assumption that all coral somatic cells can produce gametes.

Of the post-embryonic SNVs found in the sperm pools, 39.2% ± 3.5% were P+S (figure 2*b*), the same P+S SNVs as shown in figure 2*a*. An additional 52.2% ± 5.4% of SNVs in the sperm pools were found only in the sperm pool (SSPO) (figure 2*b*). A small number of sperm SNVs, 8.5% ± 2.1%, were found in all sperm pools from that colony but none of the parent samples that spawned them (labelled ASP, figure 2*b*). That two out of every five SNVs present in a given sperm pool are post-embryonic, non-germline variants indicates that the lack of an embryonic germline increases the number of SNVs in a colony's gametes by 66%, compared to what the diversity would have been if the germline were segregated at the embryonic stage. This may help explain the high degree of heterozygosity in many stony coral species, though it is not yet known what fraction of these SNVs are too deleterious to survive into adulthood.

The rate of SNVs per bp was significantly higher across the full genome than the rate of SNVs in the coding regions of the genome for all SNV types (figure 2*c*) (electronical supplementary material, table S4 for all means and Wilcoxon signed-rank test results). This may indicate that there is stronger purifying selection against SNVs in coding regions than in non-coding regions of the genome, or it may be a result of higher mismatch repair in exons [35].

We examined the spectrum of mutations, the relative proportions of mutations in different classes, and found no significant differences in spectra among parent only, shared, and germ-line-specific mutations (electronic supplementary material, figure S6). These data confirm lack of a signature of ultraviolet (UV)-associated mutations in corals^13^, which is intriguing considering that these colonies grow in high UV conditions, and in highly oxygenated warm water [36].

Losses of heterozygosity tend to arise as a result of gene conversion owing to homologous recombination, a form of double stranded DNA break repair [37]. Consistent with previous findings [12], 38.3% ± 3.0% (1 s.e.m.) of all parent SNVs being GoH and 61.7 ± 3.0% (1 s.e.m.) being LoH. SNVs that were shared by the parent branch tissues and the sperm had a much higher fraction of GoH and lower fraction of LoH (73.8 ± 3.6% and 26.2 ± 3.6%, respectively) than did parent SNVs that were not found in the sperm (25.2 ± 2.4% and 74.8 ± 2.4%, respectively) **(**Wilcoxon signed-rank test, *V* = 28, *p* = 0.015) (figure 2*d*). SNVs found in just a single sperm pool had approximately equal proportions of each type, 51.1% ± 2.5% GoH and 48.9 ± 2.5% LoH SNVs. High LoH in soma that is not inherited by the sperm could be owing to high incidence of double-strand breaks in somatic cells exposed to high light and photosynthetically derived oxidation, or high LoH levels in the PO SNVs may reflect stronger selection against GoH than LoH in differentiated somatic cells.

Because DNA was extracted from tissue scrapings encompassing multiple polyps per parent branch, we were initially concerned that the parent sample might be a heterogenous mix of somatic and germ tissues. The scraping method was intended to take off just the somatic tissue and not the germ or stem cells. To check this, for each P+S SNV, we plotted the average VAF of the mutant parent (that is, the average VAF for the two technical replicate libraries) against the average VAF of the mutant sperm pool:

$$\begin{array}{rl} \mathrm{VAF} & =\frac{\mathrm{no}.\;\mathrm{of}\;\mathrm{reads}\;\mathrm{supporting}\;\mathrm{SNV}}{\mathrm{total}\;\mathrm{no}.\;\mathrm{of}\;\mathrm{reads}\;\mathrm{at}\;\mathrm{locus}}.\\ \mathrm{Average}\;\mathrm{VAF} & =\frac{\left({\mathrm{VAF}}_{1}+{\mathrm{VAF}}_{2}\right)}{2}. \end{array}$$

**Figure 3.**
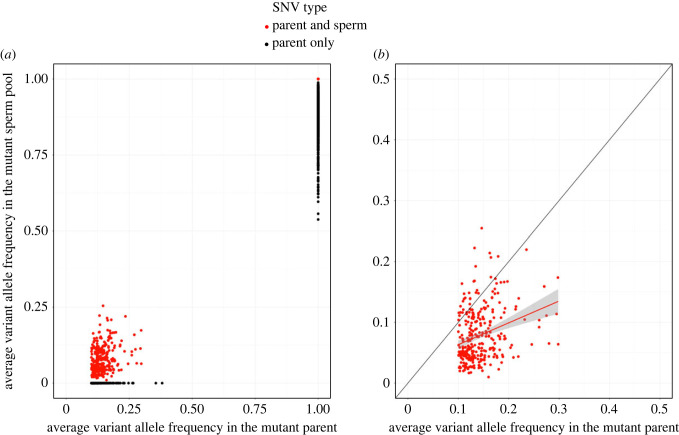
Variant allele frequencies (VAFs) for parents and their respective sperm pools. (*a*) GoH mutations for which sperm VAF =0 were classified as parent only (black), likewise LoH variants in the parent for which the VAF < 1 in the sperm were classified as parent only. All other SNVs were classified as being shared by both a parent branch and its corresponding sperm pool (red). (*b*) For GoH SNVs that are shared by a parent branch and its sperm, average VAF of the two replicate parent libraries is weakly correlated with the average VAF of the two replicate sperm pool libraries. The slope of the relationship is 0.36 and *R*^2^ = 0.081 (trendline shown in red). The black line shows a 1 : 1 line for reference.

The slope of the trendline was 0.36, with *R*^2^ = 0.081 and *p* = 5.9 × 10^−8^ (figure 3). This suggests that there may have been some undercounting of the number of mutant reads in the sperm. The distribution of average VAF in the parents for SNVs classified as ‘PO’ has a larger leftward skew, which also suggests that some SNVs found at low frequency in the parent samples may have been missed in the sperm (electronic supplementary material, figure S7). This gives us confidence that the parent samples were in fact all or almost all somatic tissue, and that the mutations found in the parents were in the soma.

**Figure 4.**
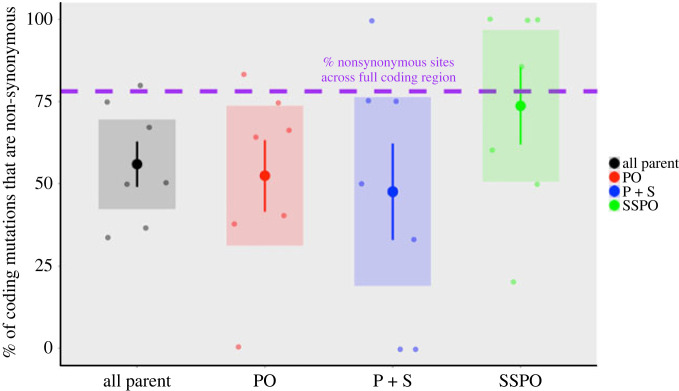
The mean per cent of protein-coding SNVs that are non-synonymous across coral branches (*n*= 7) for all parent, parent only (PO), parent and sperm (P + S) and single sperm pool only (SSPO) categories. Large circles indicate the mean, with error bars indicating ± 1 s.e.m. and shaded boxes indicating 95% confidence interval. Each coral branch (*n* = 7) is shown as a smaller point for each category.

To explore the role of selection on patterns of genome change, we compared the rates of missense and synonymous SNVs across four classes: all somatic SNVs, PO SNVs, P+S SNVs and SSPO SNVs. There were no coding SNVs in the ASP category. The average rate of coding mutations was highest in PO SNVs (6.0 × 10^−7^ ± 1.6 × 10^−7^; electronic supplementary material, figure S8). Across the full *A. hyacinthus* proteome, we estimate that 78.1% of sites are non-synonymous and 21.9% of sites are synonymous (figure 4). The per cent of coding mutations that were missense was higher in SSPO SNVs (73.7 ± 11.8%) than in the other categories (55.2 ± 6.8% all somatic, 51.5 ± 10.7% PO, 47.6 ± 14.6% P+S; figure 4). The higher mean per cent missense in SSPO SNVs was not statistically significant, probably attributable to the small number of coding mutations in each category. Like most studies on somatic mutations to date, the small number of coding mutations in this study (94) leaves us underpowered to detect selection [38]. However, the fairly consistent pattern of more missense mutations in sperm pool samples than somatic samples provides a first hint that the SNVs in the soma may experience stronger negative selection than germline SNVs.

## 4. Discussion

**Figure 5.**
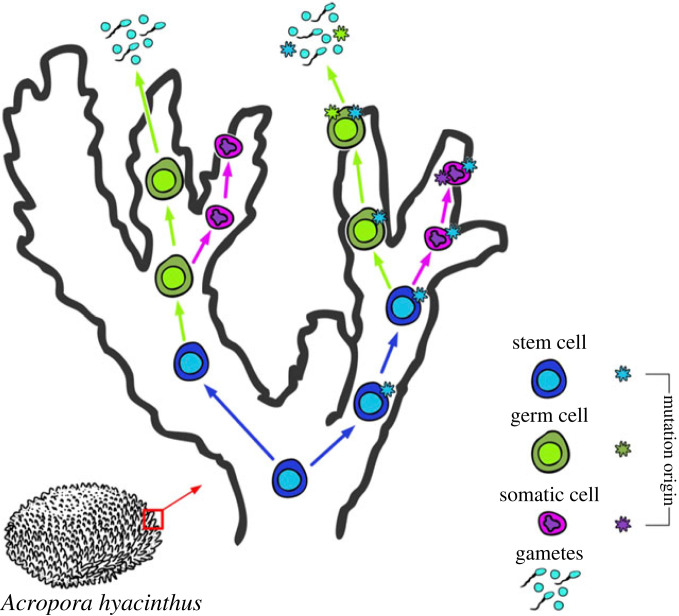
Schematic for how SNVs that arise at different stages of cell lineage development and differentiation proliferate. SNVs that arise in stem cells may be found in both germ and somatic cells later, if the mutant stem cell lineage differentiates into both germ and soma. SNVs that arise in soma or germ cell lineage post-differentiation will only be found in those differentiated lineages. Figure by Shayle Matsuda.

In a high-resolution analysis of post-embryonic mutations in corals, we found some variants only in a specific parent branch, some only in the sperm from that branch, and some that were in both. Across seven independent samples, 26% of the SNVs that we identified in parent tissue were also present in the sperm spawned from that branch, but 74% were not. If we had found only separate parent and sperm SNVs this would have shown that *Acropora* corals have classical Weismannian germ and somatic cell lineage differentiation at the embryonic stage, which has been suggested previously [17]. Likewise, if we had found that all parent tissue SNVs were also in the sperm, or that there was a strong correlation between the parent VAF and the sperm VAF for P + S SNVs, we would have concluded that *Acropora* corals developed gametes directly from those tissues [15,16]. By contrast, the occurrence of both types of patterns suggests that germ cell differentiation in corals occurs in a different manner than posited by Weismann's Germ Plasm Theory. Overall, these results suggest that post-embryonic mutations in corals occur in somatic cells after differentiation, in germ cells after differentiation, and also in a common putative multipotent stem cell line in corals that later differentiates into somatic and germ cell lines (figure 5).

We also tested other possible explanations for this complex dataset. First, if the parent samples were a mix of somatic and germ and/or stem cells, then a germline mutation may have erroneously been called a somatic mutation. If that were the case, then the VAF of the parent would be considerably lower than the VAF of the sperm pool, because the sperm pool's mutant would not be diluted by non-mutant somatic tissue. In that case, we would expect the trendline of the parent VAF : sperm VAF linear model to have a slope significantly greater than 1 (figure 3). In reality, the slope of the trendline was less than 1, suggesting that we undercounted the number of mutant reads in the sperm. This gives us confidence that the parent samples were in fact all or almost all somatic tissue, and that the mutations found in the parents were in the soma.

In addition, if only some somatic cells in a polyp were capable of producing gametes, or if the somatic tissue of a polyp was a mosaic of more than one genotype, then we would expect the VAF of the parent sample and the VAF of the sperm sample to be close to a 1 : 1 ratio, or at the very least highly correlated. That the slope of the trendline was in fact just 0.36, and that the correlation between parent and sperm VAF was low (*R*^2^ = 0.081), indicates to us that this was not a case of mosaicism within the parent soma leading to mosaicism within the sperm (figure 3). Rather, the lack of correlation indicates that sperm were produced by a source not represented by the parental VAF: a source of gamete-producing cells.

Other verified SNV types rule out the possibility that somatic cells directly produce sperm in these corals. First, we find SSPO SNVs: both GoH and LoH variants that were present in the sperm but not in the parent samples. We also find LoH SNVs that are in the parent sample, but not in the sperm. Additionally, the PO and P + S SNVs have different (almost inverse) ratios of GoH : LoH SNVs in each category, suggesting that these arise from different mechanisms and in different tissues.

Another potential cause for mosaicism among branches of a coral colony is chimerism, which results from two sexually produced genotypes fusing together. However, a chimera would be expected to have a much higher level of genetic variation than was found in this study. The average rate of occurrence of variants among branches was 1.76 ± 0.23 × 10^−6^ (1 s.e.m.) SNVs bp^−1^ branch^−1^ for PO and 0.59 ± 0.1 × 10^−6^ (1 s.e.m.) SNVs bp^−1^ branch^−1^ for P + S. These rates are orders of magnitude lower than the rate of SNP differences between two *A. hyacinthus* genotypes found on the same reef [39]. Other molecular genetic studies have identified chimeric acroporids, including *A. hyacinthus*, in the wild, but their prevalence is fairly rare at about 3% [40]. Future work tracking SNV inheritance in a chimeric colony will provide useful insight about development and reproduction in a colony that is made up of more than one sexually produced genotype.

Because we found: (i) that some SNVs are shared by sperm but the majority are not, (figure 2*a*), (ii) that there was very low correlation in SNV VAF between the somatic tissue and the sperm samples (figure 3*b*), (iii) that there were LoH SNVs in the parent branches that were not shared by the sperm (figure 2*c*), (iv) that there were SNVs found only in the sperm samples (figure 2*b,c,d*), and (v) that there were inverse ratios of GoH : LoH in the PO and P+S SNVs (figure 2*d*), we hypothesize that in colonial corals, shared parent and sperm SNVs derive from mutations in a common ancestor stem cell lineage that self-renews and proliferates through the colony, and that eventually differentiates into both germ and soma throughout the colony's adult life. We hypothesize that these cells are putative multipotent stem cells. Multipotent stem cells, including i-cells, have been described in Hydrozoan cnidarians [7,8]. Multipotent stem cells have not yet been identified in Anthozoans, although stem-like cells called amoebocytes have been described [41]. Although i-cells have not been identified in corals, cells that look like the i-cells are present in larvae of the coral *A. millepora* [42]. Whether or not Hydrozoan-like i-cells are present in corals, our data suggest that branch-specific SNVs shared in germ and somatic cells first arose in a putative stem cell lineage proliferating in that branch, and then differentiated into germ and soma (figure 5). SNVs found only in the parent but not in the sperm would have arisen in terminally-differentiated somatic cells that cannot produce gametes, and SNVs found only in the sperm would have arisen from differentiated germ cells (figure 5).

Based on our data from *A. hyacinthus*, germ and soma differentiation appear to happen locally, resulting in evident mosaicism in every branch (figures 2*a* and 5). We hypothesize that the programme of sequential germ line differentiation during adult life shown in Hydrozoans may be a conserved trait across Cnidaria and was present in the cnidarian common ancestor. Putative primordial germ cells (PGCs) have been identified in *Nematostella vectensis* [43,44], which like *A. hyacinthus* is an Anthozoan, but so far a multipotency programme like that described in Hydrozoans has not been identified. However, lack of evidence for a pluripotent lineage in *N. vectensis* does not preclude the possibility that putative PGCs in *N. vectensis* might also be capable of differentiating into somatic cells, because the same germline markers, most notably *vas2,* are expressed and functional in both PGCs and in multipotent stem cells of *Hydra* and other non-Cnidarian species [44].

The alternative hypothesis is that in early cnidarians, self-renewing epithelial cells acted like stem cells, and that the specific multipotency programme represented by i-cells is a derived trait particular to Hydrozoans [41]. Under this hypothesis, the stem-like cells in our study may be an additional type unique to anthozoans. Current studies are based on a wide range of evidence in different taxa from different datasets, and so experimental tests for multipotency in non-Hydrozoan cnidarians, as well as comparative studies of SNV inheritance across a broad array of cnidarian taxa, will be necessary to test the hypothesis that the germline multipotency programme is an ancestral cnidarian trait. In addition, the patterns of inheritance we described in the study fit the predictions made by Solana [5] for planarians which have multipotent stem cells–when stem cells can differentiate into both soma and germ cells, mutations that appear in both the soma and the germline are derived from mutations in those stem cells. Occurrence of this pattern in planaria, hydrozoans, and corals could suggest that the germline multipotency programme is an ancestral metazoan trait.

Because the putative stem cell mutations are inherited by the soma, they are tested by natural selection and the environment in a way that the germ cell-derived mutations are not. For instance, if a stem cell mutates and then that mutant stem cell line gives rise to both soma and sperm, then that mutation is exposed to the environment in the form of tentacle, gastroderm, or other somatic cell types. If the mutation is deleterious for the soma of the polyp, then the polyp will be less likely to reproduce and pass on that deleterious mutation. If the mutation is neutral or advantageous for the polyp soma, then the polyp may survive to pass on the mutation, because that mutation is also in the polyp's gametes. Note that this does not mean that there is inheritance from soma to sperm in this scenario (figure 5). Rather, the presence of the stem cell-derived mutations in the soma allowed the polyp to reproduce (or not), and because that stem cell-derived mutation was also in the gametes of the polyp, it can be inherited. This is a significant departure from the conclusions that other recent empirical papers have drawn, but it is very much in line with classical theory.

In support of the above hypothesis, we have evidence for some purifying selection on SNVs in stem and somatic cells, but not in germ cells. Germline coding SNVs showed the same rate of non-synonymous changes as are estimated to be in the full proteome, whereas the fraction was considerably below the neutral threshold for somatic and stem cell coding SNVs (figure 3). A lower fraction of non-synonymous changes suggests active filtering of SNVs by purifying selection against variants that change the amino acid sequence. Thus, our data suggest that there is little selection happening on germline cells, but there is evidence for purifying selection on somatic and stem cells. If putative stem cell SNVs are subject to selection, then the selection regime that growing stem cell lines face could select for novel beneficial changes as well as select against deleterious ones [6,45]. Reef building corals are extremely sensitive to small increases in temperature, but these environmental changes frequently result in the death of just part of a colony. If partial survival of a colony is the result of selection for post-embryonic SNVs in different parts of the colony, then adaptation to environmental change may occur over the lifetime of a single colony. If some of those post-embryonic SNVs are inherited by the surviving polyps' gametes, then this may be an alternative, rapid route to adaptation for corals.

Our data reveal a putative stem cell lineage that self-renews and remains multipotent throughout the adult lifespan in an anthozoan, the likes of which have previously been described in hydrozoans. We also show, for the first time to our knowledge, the genome-level consequences of SNVs in stem cells on the mutation load of a long-lived animal species that lacks an embryonic germline. SNVs in the stem cell lines of a coral colony increase the number of SNVs in the sperm by 66%. This may help to explain the high degree of heterozygosity and adaptive polymorphism in many stony coral species. Mechanisms that corals use to avoid mutational meltdown in long-lived cell lineages might include consistent screening by natural selection in proliferating cell lines, or yet-to-be discovered controls on coding gene mutation rates. Also, importantly in the context of global climate change, selection in proliferating cell lines may increase the frequency of heritable adaptive polymorphisms in some stony coral species.

## Data Availability

All raw fastq files, as well as the *Acropora hyacinthus* genome version 1 assembly, are accessioned under BioProject PRJNA707502 at NCBI. The accession numbers for the fastqs are SAMN18207983-SAMN18208014 and the accession number for the assembly FASTA is SAMN20335437. The code and data files used for this study can be found at https://github.com/eloralopez/CoralGermline. Data is also provided in the electronic supplementary material [46].
